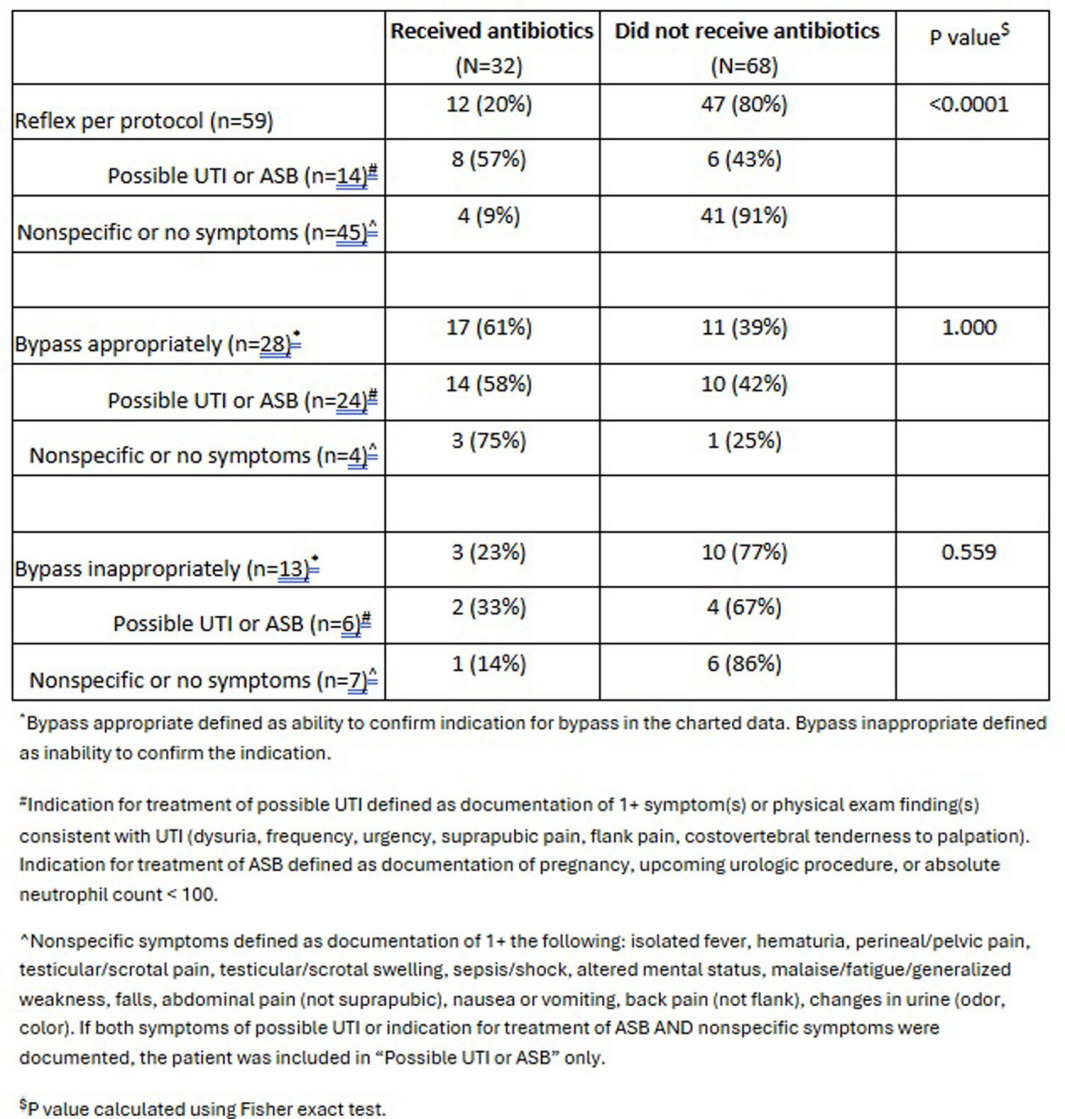# 211 Exploring Perceptions of Healthcare Workers Across the Globe Regarding Antimicrobial Stewardship Training Courses

**DOI:** 10.1017/ash.2026.10443

**Published:** 2026-06-23

**Authors:** Megan Uehling, Milner Staub, Barbara Mora, Lauren Johansen, Sharon Onguti, Rebecca Stern

**Affiliations:** 1 Vanderbilt University Medical Center; 2 Vanderbilt University

## Abstract

**Background:** Over-utilization and inappropriate interpretation of urinalyses and urine dipsticks drives unnecessary urine cultures, leading to unnecessary treatment of asymptomatic bacteriuria (ASB), avoidable antibiotic associated adverse drug effects, and risk of antimicrobial resistance. The urinalysis with reflex order set (UA w/ reflex) is a stewardship intervention to reduce unnecessary cultures. Providers can elect to bypass the reflex criteria by selecting specific clinical indications (e.g., pregnancy) to culture urine regardless of urinalysis results. We aimed to assess the appropriateness of provider bypass selection and the effect on appropriate antibiotic prescribing for urinary tract infections (UTIs). **Methods:** From a dataset of adult outpatient encounters in which the UA w/ reflex order set was used at Vanderbilt University Medical Center (VUMC) between June 1, 2022 and June 30, 2024, 120 patients were randomly selected for review as part of an ongoing study. Of those, patients <16 years of age, hospitalized within 2 days of UA, with delay of UA acquisition, or with incomplete encounter documentation were excluded (N=20). Patient and provider demographics, patient symptoms, physical exam findings, urine diagnostic testing, and antibiotics prescribed were extracted from the electronic medical record (EMR) and via manual chart review and analyzed. **Results:** Of the 100 included patients, the average age was 48.9 years and 64% were female. Of the 32 (32%) who received antibiotics, 24 (75%) had a possible UTI or indication for treatment of ASB (“appropriate”), and 8 (25%) had nonspecific or no symptoms (“inappropriate”). Among 13 patient encounters which inappropriately bypassed the reflex criteria, 1 (14%) received “inappropriate” antibiotics compared to 2 (33%) who received “appropriate” antibiotics (p = 0.559), see Table 1. Among those where the culture reflexed per protocol (n = 59), 45 had non-specific or no symptoms (76%) and 41 (91%) did not receive unnecessary antibiotics (p<0.0001). This difference was not seen in bypass appropriate or bypass inappropriate groups (p=1.00 and p=0.559 respectively), see Table 1. **Conclusions:** In this preliminary analysis, we observed high (~1/3) rates of inaccurate selection of bypass criteria; however, this was not associated with increased inappropriate antibiotic prescribing, which may have been due to the small sample size. Antibiotic prescribing was lowest when the reflex order was used appropriately. Additionally, high rates of orders without UTI symptoms were noted, suggesting potential diagnostic mis-stewardship. Behavioral drivers and clinical triggers for ordering UA w/ reflex need to be better understood to target reducing inappropriate testing.

**Figure:**